# Enhanced IL-6/phosphorylated STAT3 signaling is related to the imbalance of circulating T follicular helper/T follicular regulatory cells in patients with rheumatoid arthritis

**DOI:** 10.1186/s13075-018-1690-0

**Published:** 2018-08-29

**Authors:** Qian Niu, Zhuo-chun Huang, Xiao-juan Wu, Ya-xiong Jin, Yun-fei An, Ya-mei Li, Huan Xu, Bin Yang, Lan-lan Wang

**Affiliations:** Department of Laboratory Medicine, West China Hospital, Sichuan University, 37#, Guoxue Alley, Chengdu, 610041 China

**Keywords:** Rheumatoid arthritis, Follicular helper T cells, Follicular regulatory T cells, PD-1, ICOS, IL-21, IL-6, STAT3, DAS28-CRP

## Abstract

**Background:**

Follicular helper T (Tfh) cells are specialized in helping B lymphocytes, which play a central role in autoimmune diseases that have a major B cell component, such as in rheumatoid arthritis (RA). Follicular regulatory T (Tfr) cells control the over-activation of Tfh and B cells in germinal centers. Dysregulation of Tfh cells and Tfr cells has been reported to be involved in the pathogenesis of some autoimmune diseases. However, the balance of Tfh and Tfr cells, and their roles in the development and progression of RA are still not clear.

**Methods:**

In this study, we enrolled 44 patients with RA (20 patients with active RA and 24 patients with inactive RA) and 20 healthy controls, and analyzed the frequencies of circulating Tfh and Tfr cells, expression of programmed death-1 (PD-1), inducible co-stimulator (ICOS), intracellular IL-21, and pSTAT3 in Tfh cells, and serum levels of IL-6. The correlation among these parameters and that of Tfh or Tfr cells with disease activity were also analyzed.

**Results:**

Patients with RA (especially active RA) had higher frequencies of Tfh cells, but lower percentages of Tfr cells, thereby resulting in elevated ratios of Tfh/Tfr. Expression levels of PD-1 and IL-21 in Tfh cells were higher in patients with RA than in healthy subjects, while no difference in ICOS expression was observed between patients and controls. Both pSTAT3 expression and serum IL-6 levels increased in patients with RA, and positive correlation between them was observed. Additionally, pSTAT3 expression was positively correlated with Tfh cell frequency. The Disease Activity Score in 28 joints based on C-reactive protein (DAS28-CRP) was negatively correlated with Tfr cell frequency, but was positively correlated with both Tfh/Tfr ratio and PD-1 expression.

**Conclusions:**

Results demonstrated that enhanced IL-6/pSTAT3 signaling may contribute to promotion of Tfh cells, consequently skewing the ratio of Tfh to Tfr cells, which may be crucial for disease progression in RA.

**Electronic supplementary material:**

The online version of this article (10.1186/s13075-018-1690-0) contains supplementary material, which is available to authorized users.

## Background

Rheumatoid arthritis (RA) is a chronic inflammatory autoimmune disease, characterized by symmetrical inflammation of synovium, which mainly affects the peripheral diarthrodial joints, leading to progressive destruction of articular cartilage and bones, culminating in severe pain and disability [1]. The pathogenesis of RA is complicated and not yet fully elucidated; however, both innate mechanism and highly evolved adaptive immune functions seem to operate simultaneously to create and propagate the inflammatory reactions attacking the joints [2, 3]. The robust production of autoantibodies, including rheumatoid factor (RF) and anti-cyclic citrullinated peptides antibodies (ACPA), is a crucial factor in RA pathophysiology, that can lead to immune-complex deposition and the subsequent recruitment and activation of inflammatory leukocytes in the joints [4]. Usually, it is preceded by activation, somatic diversification, and affinity maturation of auto-reactive B lymphocytes, which occur in the germinal centers (GC) [5]. In these processes, follicular helper T (Tfh) cells are the principal CD4^+^ T helper cell subpopulation, providing essential help to B cells [6], whereas regulatory T cells (Tregs) function to control B cell responses and play a critical role in the establishment of self-tolerance.

Tfh cells occur predominantly in the B cell follicles, playing a key role in GC formation, B cell development and maturation, and immunoglobulin class switching. They can be distinguished from other subsets of differentiated CD4^+^ T cell lineages by the high expression of C-X-C chemokine receptor type 5 (CXCR5), programmed death-1 (PD-1), inducible co-stimulator (ICOS), CD40 ligand (CD40L), and secretion of interleukin-21 (IL-21) [7, 8]. Accumulating evidence has shown that dysregulation of Tfh cells and IL-21 could result in disordered autoimmunity, contributing to various autoimmune diseases, such as systemic lupus erythematosus (SLE) and ankylosing spondylitis (AS) [9, 10]. The high frequency of circulating Tfh cells (which could imply enhanced cell differentiation) is also reported to correlate with disease activity in patients with new-onset RA [11]. Tfh cells are localized in lymphoid follicles, making it difficult to study these cells. However, a recent study has shown that human peripheral blood CD4^+^CXCR5^+^ cells have functional properties similar to Tfh cells [12]. Circulating CXCR5^+^ T cells, which secrete high levels of IL-21 to promote B cell differentiation into plasma cells, are more capable of facilitating B cell maturation and humoral responses than CXCR5^−^ T cells [13]. Thus, circulating Tfh cells are crucial for the pathogenesis of autoimmune diseases [14].

Tfh cell differentiation involves multiple micro-environmental factors; one clinical study showed that secretion of IL-6 by plasmablasts resulted in Tfh cell differentiation [15]. IL-6 is involved in human Tfh cell differentiation; after binding to its receptor, it phosphorylates the signal transducer and activator of transcription 3 (STAT3), which is essential for dimerization and nuclear translocation [16]. IL-6 is an inflammatory cytokine with an important role in the pathogenesis of RA and our previous study [17] had shown that serum IL-6 level obviously increased in patients with RA. In this study, we hypothesized that the circulating Tfh cells may be elevated via the IL-6/STAT3 signal pathway.

Enhanced GC reactions must be regulated to prevent the excessive production of auto-antibodies, and an increasing number of studies have found that follicular regulatory T (Tfr) cells—a specialized population of Tregs that are primarily located in germinal centers—can specifically suppress Tfh and B cells to control the GC reaction [18, 19]. Tfr cells share the phenotypic characteristics of both Tfh cells and classical Tregs, simultaneously expressing Foxp3 and CXCR5, as well as PD-1 and ICOS [18–20], thereby playing an opposing role with Tfh cells in the regulation of humoral immunity [21]. The immune homeostasis of Tfh and Tfr cells is reported to be disrupted in the peripheral blood of patients with autoimmune diseases such as SLE, myasthenia gravis (MG), and multiple sclerosis (MS) [22–24]. However, few studies have investigated the role of Tfh/Tfr imbalance in the pathogenesis of RA in detail. The role of Tfr cells and their relationship with other T cell subsets in the abnormal immune responses of RA remain to be elucidated.

Therefore, we determined the frequencies of circulating CD4^+^CXCR5^+^Foxp3^−^ Tfh and CD4^+^CXCR5^+^Foxp3^+^ Tfr cells, and the expression of PD-1, ICOS, intracellular IL-21 and phosphorylated STAT3 (pSTAT3) in Tfh cells in patients with RA and corresponding healthy controls (HCs). The correlation of Tfh and Tfr cells with disease activity in RA was investigated.

## Methods

This prospective study was performed in accordance with a protocol approved by the Ethics Committees of West China Hospital. Written informed consent was obtained from all participants.

### Participants

Forty-four patients with RA were enrolled in this study. All patients met the American College of Rheumatology (ACR)/European League Against Rheumatism (EULAR) classification criteria (2010) [25]. The exclusion criteria were as follows: subjects with other autoimmune diseases or tumors, plasma exchanges or thymectomy prior to the study, and with acute inflammation in the preceding 4 weeks. Disease activity was assessed by the Disease Activity Score in 28 joints based on C-reactive protein (DAS28-CRP). Routine measurements were made of CRP, RF, and ACPA (screened by electro-chemiluminescence immunoassay (ECLIA) using the Cobas e601, Roche Pharma Itd., Reinach, Switzerland). The patient group was compared to a group of 20 age-matched and sex-matched HCs. The characteristics of the patients and HCs are shown in Table 1.

**Table 1:** Characteristics of the patients with RA and the healthy controls

Characteristics	Active RA (*n* = 20)	Inactive RA (*n* = 24)	HCs (*n* = 20)	*P* value
Age (years)^a^	46 (38–57)	47 (40–53)	48 (39–55)	> 0.05
Female (*n* (%))	16 (80.0)	18 (75.0)	15 (75.0)	> 0.05
Symptom duration (months)^a^	10 (5–60)	15 (8–70)	ND	0.015^b^
Swollen joint count (out of 28)^a^	5 (3–8)	2 (1–5)	ND	0.039^b^
Tender joint count (out of 28)^a^	6 (2–9)	4 (1–7)	ND	0.027^b^
CRP (mg/L)^a^	6.5 (2.1–13.8)	4.0 (1.4–9.2)	ND	0.031^b^
DAS28-CRP (3)^a^	4.2 (3.5–5.6)	1.8 (1.0–2.8)	ND	0.001^b^
ACPA (IU/ml)^a^	451.9 (222.2–1208.0)	129.6 (6.0–322.6)	ND	0.006^b^
RF (IU/ml)^a^	132.0 (30.73–267.3)	23.8 (19.0–75.1)	ND	0.027^b^
ACPA ≥ 17 IU/ml (*n* (%))	18 (90.0)	17 (70.8)	ND	> 0.05^b^
RF ≥ 20 IU/ml (*n* (%))	15 (75.0)	17 (70.8)	ND	> 0.05^b^
ACPA ≥ 17 IU/ml + RF ≥ 20 IU/ml (*n* (%))	14 (70.0)	16 (66.7)	ND	> 0.05^b^
ACPA ≤ 17 IU/ml + RF ≤ 20 IU/ml (*n* (%))	3 (15.0)	4 (16.7)	ND	> 0.05^b^

*RA* rheumatoid arthritis, *HC* healthy controls, *CRP* C-reactive protein, *DAS28* Disease Activity Score 28, *ACPA* anti-cyclic citrullinated peptide antibody, *RF* rheumatoid factor, *ND* not determined

^a^Data are presented as median (IQR)

^b^Patients with active RA vs. patients with inactive RA, Mann–Whitney *U* test

### Cell preparation

The experiments were carried out within 1 hour of obtaining the heparinized venous blood samples from the participants. For analysis of intracellular IL-21, 500 μl of whole blood from every sample was cultured in a complete culture medium (Roswell Park Memorial Institute (RPMI) 1640 supplemented with 10% heat-inactivated fetal calf serum) for 5 h, in the presence of phorbol 12-myristate 13-acetate (PMA, 50 ng/ml, Sigma-Aldrich, St. Louis, MO, USA), ionomycin, calcium salt (1 μg/ml, Sigma-Aldrich), and monensin (BD GolgiStop™, 1 μg/ml, BD Biosciences, San Diego, CA, USA). The incubators were set at 37 °C under a 5% CO_2_ environment. The remaining unstimulated whole blood was aliquoted into tubes (50 μl each) for further analysis of PD-1, ICOS, Tfr, and pSTAT3.

### Flow cytometry

The monoclonal antibodies targeting human CD3 (clone SP34–2, peridin chlorophyll protein (PerCP)), CD4 (clone SK3, fluorescein isothiocyanate (FITC)), IL-21 (clone 3A3-N2.1, phycoerythrin (PE)), and pSTAT3 (clone 4/P-STAT3, PE) were all purchased from BD Biosciences; PD-1 (clone MIH4, PE), ICOS (clone ISA-3, PE), and Foxp3 (clone 236A/E7, PE) were all from eBioscience (San Diego, CA, USA); and CXCR5 (clone J252D4, APC) was from BioLegend (San Diego, CA, USA). Appropriate isotype controls were used to enable correct compensation and confirm antibody specificity. For PD-1 and ICOS analysis, 50 μl of unstimulated cells were incubated with surface-staining antibodies (CD3-PerCP, CD4-FITC, CXCR5- allophycocyanin (APC), and PD-1-PE or ICOS-PE) at 4 °C for 30 min in the dark. To detect intracellular IL-21 and Tfr cells (defined as CD4^+^ CXCR5^+^Foxp3^+^ T cells), 50 μl stimulated (for IL-21 detection) and 50 μl unstimulated cells (for Tfr detection) were incubated with surface-staining antibodies (CD3-PerCP, CD4-FITC, and CXCR5-APC) at 4 °C for 30 min in the dark. Surface-stained cells were fixed and permeabilized with a Foxp3 Staining Set (eBioscience) and stained with PE-conjugated IL-21 or PE-conjugated Foxp3. For pSTAT3 analysis, 50 μl of unstimulated cells were incubated with surface-staining antibodies (CD4-FITC and CXCR5-APC) at 4 °C for 30 min in the dark. Then, 20 μg/ml recombinant human (rh) IL-6 (MN 550071) was added to stimulate the 50-μl surface-stained whole blood for 30 min at 37 °C in the dark. Stimulated cells were lysed and fixed with Lyse/Fix buffer (BD Biosciences) at 37 °C for 10 min and then permeabilized in Perm Buffer III (BD Biosciences) for 30 min on ice. Finally, the cells were stained with PE-conjugated pSTAT3 (pY705) and PerCP-conjugated CD3 at 4 °C for 30 min in the dark, after washing twice with BD Pharmingen stain BSA buffer (BD Biosciences).

Stained cells were run on a FACSCanto II cytometer (BD Biosciences), and the data were analyzed using FACSDiva software (BD Biosciences).

### Determination of serum IL-6

Serum samples were collected on the day of flow cytometry and stored at − 80 °C before detection. The concentration of serum IL-6 was quantified by ECLIA using the Cobas e601 (Roche) instrument, following the standard operating procedure (SOP) of the Department of Laboratory Medicine in West China Hospital of Sichuan University. Results are expressed as picograms per milliliter.

### Statistical analysis

Summary statistics (number and percentage or median and interquartile range (IQR)) were used to describe the participants’ baseline characteristics. Numerical results were expressed as mean ± SEM or median (IQR), and analyzed using the IBM SPSS software (version 22.0; IBM Corp., Armonk, NY, USA). The significance level was set at 0.05 for all statistical tests. The data were initially analyzed using analysis of variance or the Kruskal-Wallis H test. If a significant result was observed, Holm-Sidak’s test or Dunn’s test was used to detect inter-group differences. Spearman’s correlation coefficient with the two-tailed *P* value was calculated to test for correlation between pairs of continuous variables.

## Results

### Frequencies of circulating Tfh and Tfr cells in patients with RA and the HCs

To investigate the status of circulating Tfh and Tfr cells, we detected the frequencies of CD4^+^CXCR5^+^Foxp3^−^ Tfh and CD4^+^CXCR5^+^Foxp3^+^ Tfr cells, which were gated from CD3^+^CD4^+^ T cells in a flow cytometry analysis of patients with RA and HCs (Additional file 1: Figure S1A, B). According to the DAS28-CRP score, patients with RA were divided into two groups: the active RA group (*n* = 20) with DAS28-CRP > 3.2 and the inactive RA group (*n* = 24) with DAS28-CRP ≤ 3.2.

**Fig. 1:**
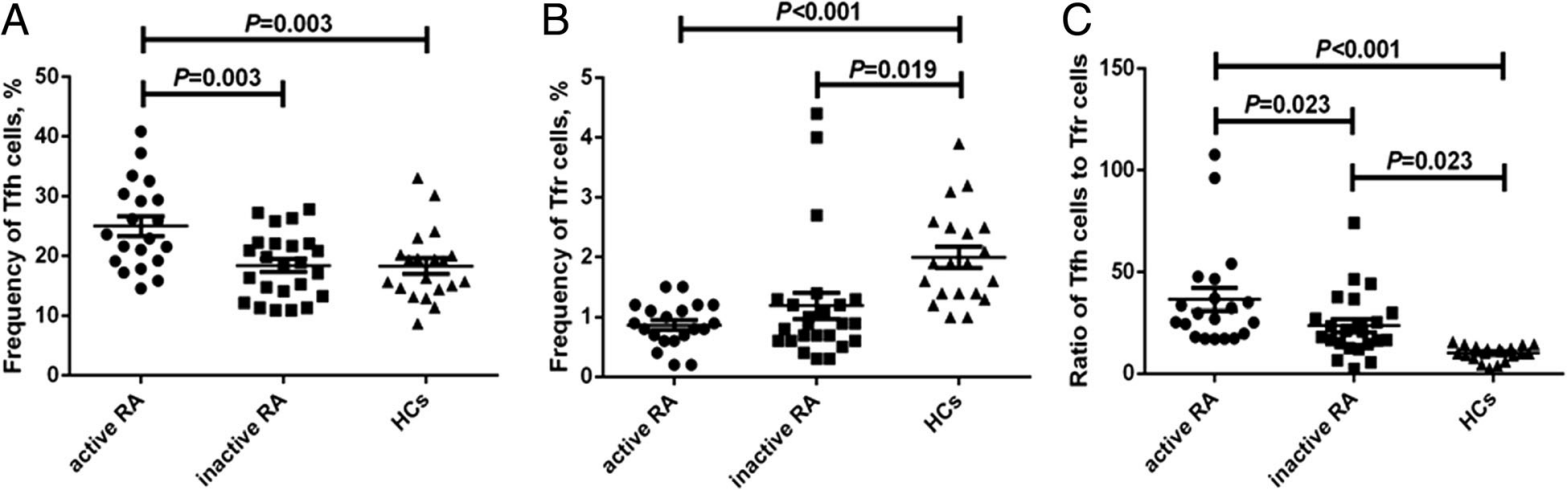
Frequencies of circulating follicular helper T (Tfh) and follicular regulatory T (Tfr) cells in patients with rheumatoid arthritis (RA) and the healthy controls (HCs). Horizontal bars indicate the mean and error bars represent the SEM. The frequencies of circulating CD4^+^CXCR5^+^Foxp3^−^ Tfh cells (**a**) and CD4^+^CXCR5^+^Foxp3^+^ Tfr cells (**b**) were investigated gating on CD4^+^ T cells in patients with active RA (circles), patients with inactive RA (squares), and the HCs (triangles). **c** Ratios of Tfh to Tfr cells in patients with active RA (circles), patients with inactive RA (squares), and the HCs (triangles)

Frequencies of circulating Tfh cells in patients with active RA (25.0 ± 1.6%) were significantly higher than those either in patients with inactive RA (18.4 ± 1.1%) or in HCs (18.2 ± 1.3%) (*P* < 0.01, Fig. 1a). Compared to the HC group (2.0 ± 0.2%), frequencies of peripheral Tfr cells were significantly lower in both the active RA (0.9 ± 0.1%) and inactive RA groups (1.2 ± 0.2%) (*P* < 0.05). However, there was no obvious difference between the two RA groups (*P* > 0.05) (Fig. 1b). In addition, the active RA group had the highest ratios of Tfh cells to Tfr cells (36.5 ± 5.6%), followed by the inactive RA group (23.6 ± 3.2%) and the control group (10.2 ± 0.8%) (*P* < 0.05, Fig. 1c).

### Expression of PD-1, ICOS, intracellular IL-21 or pSTAT3 in circulating Tfh cells of patients with RA and the HCs

**Fig. 2:**
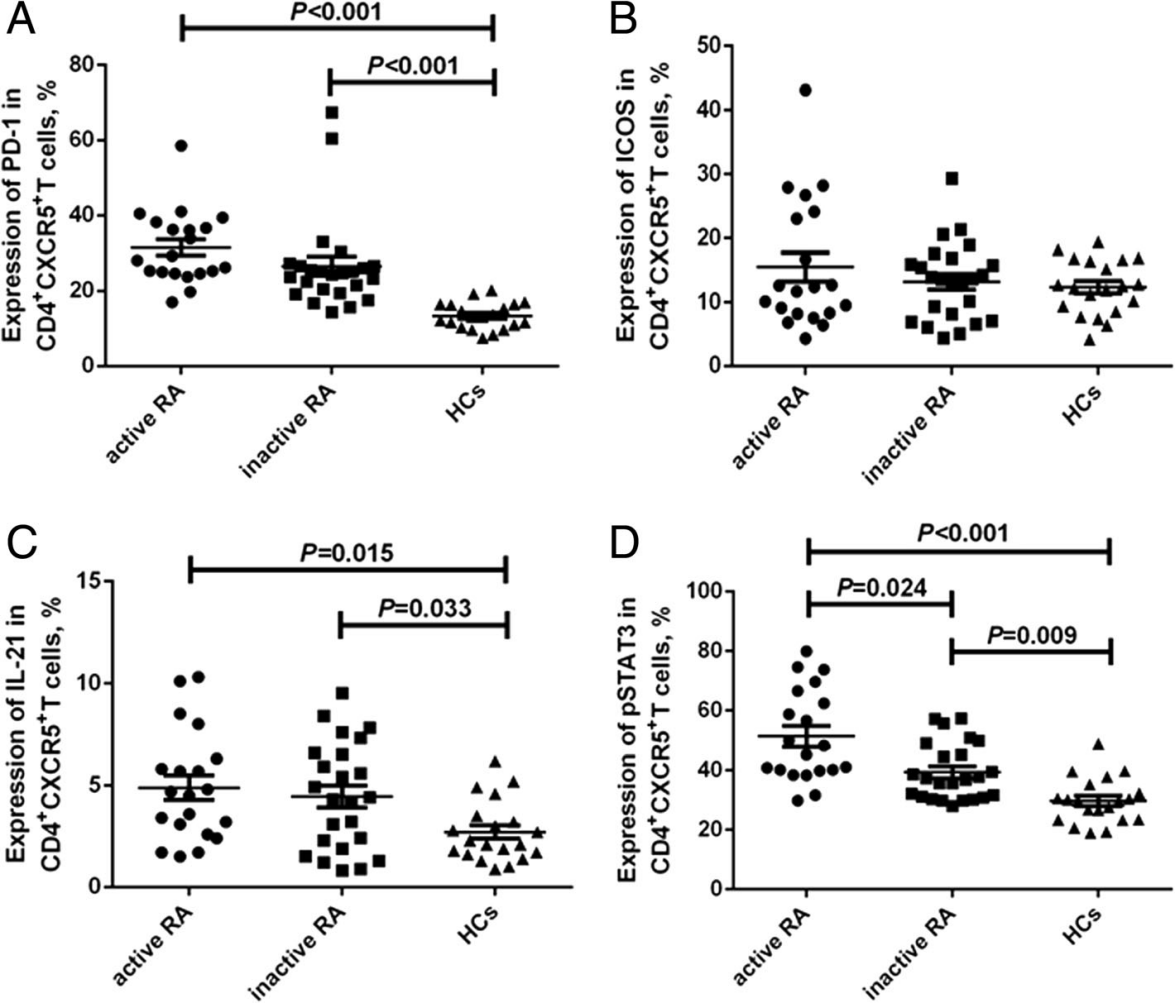
Expression of programmed death-1 (PD-1), ICOS, intracellular IL-21 or pSTAT3 in circulating CD4^+^CXCR5^+^ follicular helper T (Tfh) cells of patients with rheumatoid arthritis (RA) and the healthy controls (HCs). Horizontal bars indicate the mean and error bars represent the SEM. The expression of PD-1 (**a**), inducible co-stimulator (ICOS) (**b**), intracellular IL-21 (**c**) or phosphorylated STAT3 (pSTAT3) (**d**) was investigated gating on CD4^+^CXCR5^+^ T cells in patients with active RA (circles), patients with inactive RA (squares), and the HCs (triangles)

CD4^+^CXCR5^+^ T cell gating allowed us to investigate the expression of PD-1, ICOS, IL-21, or pSTAT3 (Additional file 2: Figure S2A-D). As shown in Fig. 2, the expression of PD-1 and IL-21 in either the active RA group (31.5 ± 2.1% and 4.9 ± 0.6%, respectively) or inactive RA group (26.5 ± 2.5% and 4.5 ± 0.5%, respectively) was significantly higher than that in the HCs (13.4 ± 0.8% and 2.7 ± 0.3%, respectively) (*P* < 0.05, Fig. 2a and c), while there was no difference observed in ICOS expression in circulating CD4^+^CXCR5^+^ T cells among the three groups (active RA, 15.5 ± 2.3%; inactive RA, 13.2 ± 1.2%; HCs, 12.3 ± 1.0%; *P* > 0.05, Fig. 2b). The expression of pSTAT3 in CD4^+^CXCR5^+^ Tfh cells was highest in the active RA group (51.3 ± 3.4%), followed by that in the inactive RA group (39.3 ± 1.9%) and the control group (29.7 ± 1.7%) (*P* < 0.05, Fig. 2d).

### Concentration of serum IL-6 in patients with RA and the HCs

**Fig. 3:**
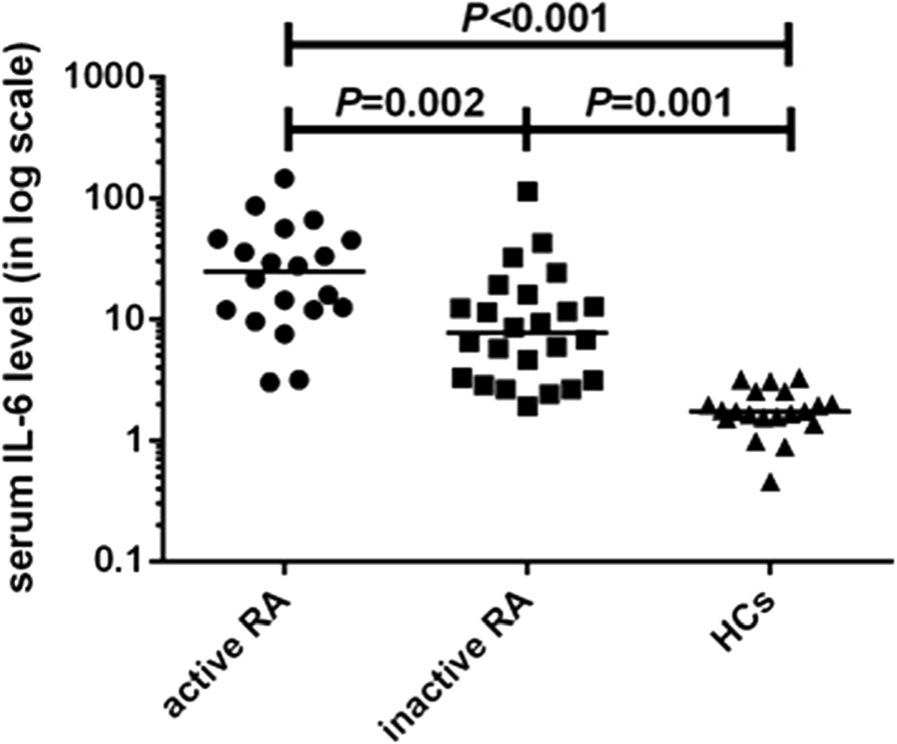
Serum IL-6 levels in patients with active rheumatoid arthritis (RA) (circles), patients with inactive RA (squares), and the healthy controls (HCs) (triangles). IL-6 levels (pg/ml) are shown in a log scale. Horizontal bars indicate the median

Significantly elevated serum IL-6 levels were observed in patients with RA compared with the control group (1.7 (1.6–2.6) pg/ml, *P* < 0.001), and the difference in levels in the two RA groups (active RA group, 27.6 (12.0–51.4) pg/ml; inactive RA group, 7.7 (3.2–15.0) pg/ml) was also significant (*P* < 0.01) (Fig. 3, IL-6 levels are shown on a logarithmic scale).

### Correlation between pSTAT3 expression and serum IL-6 level

**Fig. 4:**
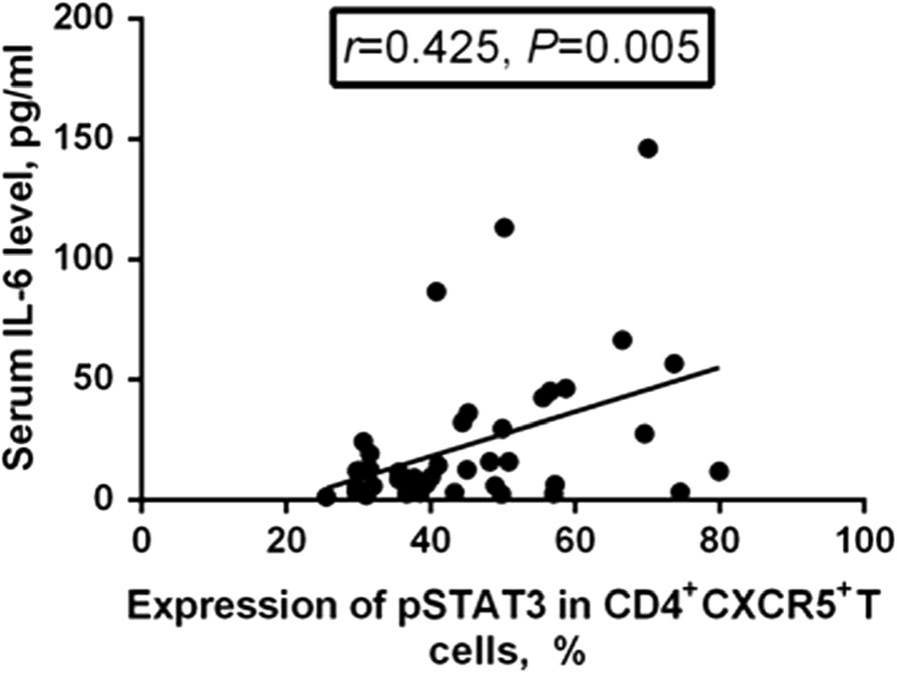
Correlation between phosphorylated STAT3 (pSTAT3) expression and serum IL-6 level. The expression of pSTAT3 in CD4^+^CXCR5^+^ follicular helper T (Tfh) cells was positively correlated with serum IL-6 level (*r* = 0.425, *P* = 0.005)

There was positive correlation between pSTAT3 expression and serum IL-6 level (*r* = 0.425, *P* = 0.005, Fig. 4).

### Correlation between pSTAT3 expression and circulating Tfh or Tfr cell frequency

**Fig. 5:**
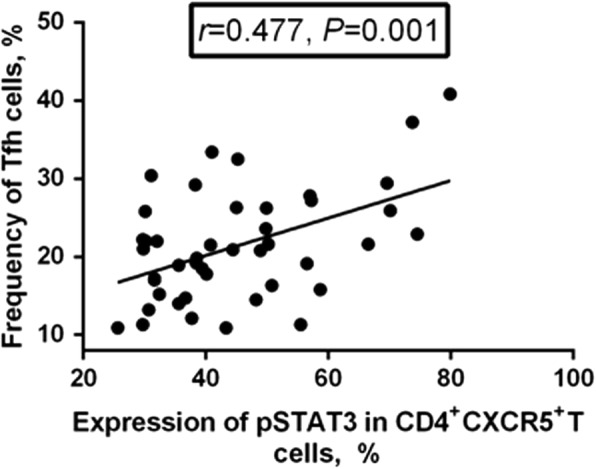
Correlation between phosphorylated STAT3 (pSTAT3) expression and circulating follicular helper T (Tfh) cell frequency. The expression of pSTAT3 in CD4^+^CXCR5^+^ Tfh cells was positively correlated with the frequency of circulating Tfh cells (*r* = 0.477, *P* = 0.001)

As shown in Fig. 5, there was positive correlation between pSTAT3 expression and circulating Tfh cell frequency (*r* = 0.477, *P* = 0.001). However, there was no correlation between pSTAT3 expression and either Tfr frequency or Tfh/Tfr ratio (*P* > 0.05).

### Correlation between the frequencies of circulating Tfh or Tfr cells and the DAS28-CRP 

**Fig. 6:**
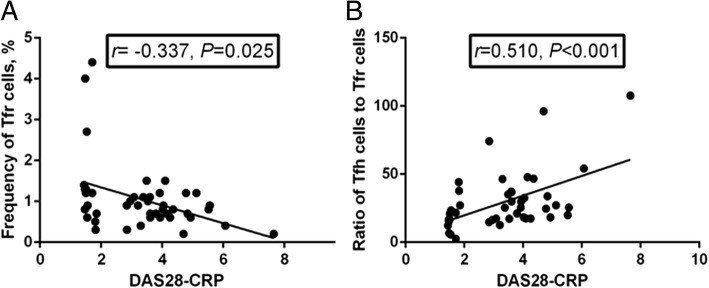
Correlation between circulating follicular helper T (Tfh) or follicular regulatory T (Tfr) cell frequency and the Disease Activity Score in 28 joints based on C-reactive protein (DAS28-CRP). The circulating Tfr cell frequency was negatively correlated with DAS28-CRP (*r* = − 0.337, *P* = 0.025) (**a**), while the Tfh/Tfr ratio was positively correlated with DAS28-CRP (*r* = 0.510, *P* < 0.001) (**b**)

The frequency of circulating Tfr cells was negatively correlated with the DAS28-CRP (*r* = − 0.337, *P* = 0.025, Fig. 6a), while the ratio of Tfh cells to Tfr cells was positively correlated with the DAS28-CRP (*r* = 0.510, *P* < 0.001, Fig. 6b). However, there was no correlation between Tfh frequency and the DAS28-CRP (*P* > 0.05).

### Correlation between PD-1, ICOS, or IL-21 expression and the DAS28-CRP

**Fig. 7:**
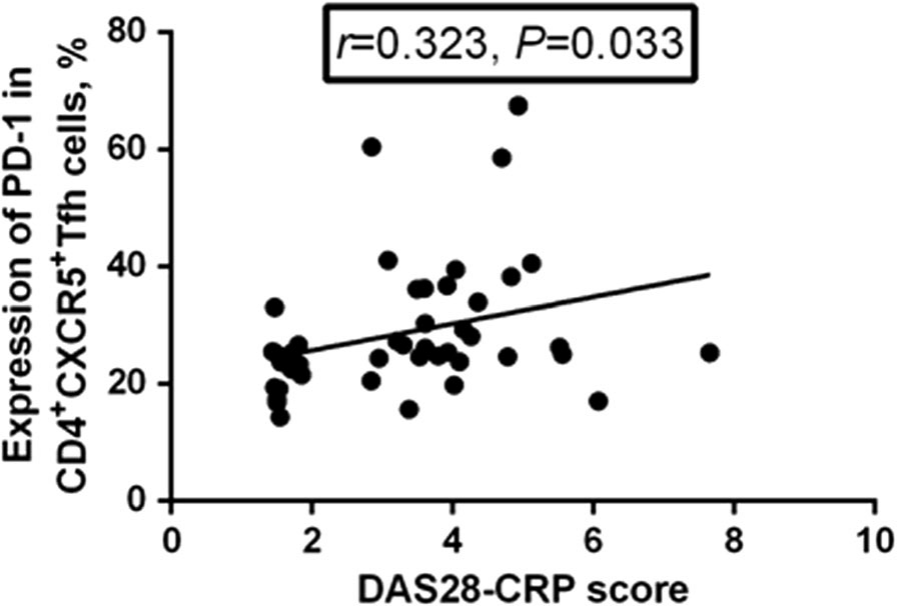
Correlation between programmed death-1 (PD-1) expression and the Disease Activity Score in 28 joints based on C-reactive protein (DAS28-CRP). The expression of PD-1 in CD4^+^CXCR5^+^ follicular helper T (Tfh) cells was positively correlated with DAS28-CRP (*r* = 0.323, *P* = 0.033)

PD-1 expression was positively correlated with the DAS28-CRP (*r* = 0.323, *P* = 0.033, Fig. 7), while neither ICOS expression nor IL-21 expression were correlated with the DAS28-CRP (*P* > 0.05).

## Discussion

Blood CD4^+^CXCR5^+^ T cells may, to some extent, represent the circulating counter-part of memory Tfh cells [13], and additional molecular markers, i.e., PD-1 and ICOS, could be used to identify the activation status of Tfh cells [6]. PD-1 supports the survival of B cells and formation of plasma cells by interacting with PD-L1 and PD-L2 on GC B cells [26], whereas ICOS can positively regulate humoral responses and IL-21 production [27]. In this study, we revealed that in both the active and inactive RA groups, the expression of PD-1 in Tfh cells was significantly higher than that in the HC group, and was positively correlated with the DAS28-CRP score. Although the expression of ICOS in Tfh cells was a little higher in patients with RA than in HCs, the difference was not significant. These findings suggest that enhanced expression of PD-1, but not ICOS, in Tfh cells might be involved in the development and progression of RA, and be of potential value as an indicator and therapeutic target of RA.

However, the inhibitory function of PD-1 on T cells and B cells, which is important in peripheral tolerance, cannot be ignored [28]. The up-regulated expression of PD-1 in Tfh cells in patients with RA, as observed in this study, might result from spontaneous compensatory regulation of the immune system so that PD-1^+^ Tfh cells can negatively regulate autoimmune responses in patients with RA. Actually, increased expression of PD-1 has been detected in human synovial tissue and fluid in RA, which might reflect the negative feedback regulation of inflammation in the joints [29]. Therefore, elucidation of the influence of enhanced PD-1 expression in Tfh cells in the development of RA would require further study.

Tfh cells secrete IL-21, a cytokine that has been found to not only promote naive T cell differentiation into potent B helper cells, but also to promote GC B cell responses through CD-40 L/CD-40 interaction [14]. Moreover, Tfh cells regulate B cell proliferation, differentiation, and antibody production via the secretion of IL-21 [13, 30]. To further understand the ability of peripheral Tfh cells to produce IL-21, we detected the expression of intracellular IL-21 in Tfh cells in patients with RA. The results showed that the expression of IL-21 in Tfh cells was significantly higher in patients with RA than in HCs, but was not correlated with the DAS28-CRP, indicating the augmented capacity of circulating Tfh cells to secrete IL-21 in patients with RA and increased potency to promote B cell proliferation, differentiation, and antibody production. Reportedly, exposure of CD4^+^ T cells to IL-21 drives them to differentiate into a Tfh cell subset partly through modulation of the expression of CXCR5 and CCR7 by IL-21 in an autocrine manner [31, 32]. However, there was no correlation between IL-21 expression with Tfh cell frequency in the present study. Actually, in addition to IL-21, numerous other cytokines, including IL-6, IL-12, and TGF-β, also reportedly contribute to Tfh cell differentiation [33].

Tfr cells express Foxp3 and have been identified to be involved in regulating the GC reaction [19]. Given that Tfh and Tfr cells have opposite roles in regulating GC responses, balance of their activities is critical for immune homeostasis. An impaired Tfr compartment could enhance Tfh activity, resulting in the expansion of auto-reactive B cells and autoantibody production [34]. The dysregulation of Tfh and Tfr cells has been reported to contribute to the development of many autoimmune diseases, including experimental autoimmune myasthenia gravis (EAMG) [35] and multiple sclerosis [24]. In this study, we presented evidence of imbalances between the circulating Tfh cell subsets and Tfr cells in patients with RA. Inconsistent with the report by Pandya JM et al. [36], we found a significant decrease in Tfr cell frequency in patients with RA, while Pandya JM et al. did not identify a significant difference in Tfr cell frequency between patients with early RA and HCs. The inconsistency between the results of Pandya JM et al. and ours might be attributed to the different symptom duration in the patients with RA enrolled in this study compared to those in the previous study. The median symptom duration in all included patients with RA was 13 months in this study, whereas it was 6 months (suggesting a very early stage of RA) in Pandya’ s study.

Patients with active RA had marginally lower Tfr cell frequency than patients with inactive RA, but the difference was not significant. In spite of this, negative correlation was identified between the frequency of circulating Tfr cells and disease activity (DAS28-CRP). With the increased proportion of Tfh subsets and decreased percentage of Tfr cells, ratios of Tfh/Tfr were significantly increased in patients with RA, although no differences in the ratios were found between patients with active RA and those with inactive RA. In addition, Tfh/Tfr ratio was positively correlated with the DAS28-CRP. These results suggested that the imbalance of Tfh and Tfr cells might contribute to the breakdown of self-tolerance in RA, thus serving as a potential target for therapy and a meaningful tool for disease evaluation.

STAT3 has been reported as a positive regulator of Tfh cell differentiation. Mice with STAT3 deficiency or humans with functional STAT3 impairment have been reported to have reduced numbers of Tfh cells and an attenuated B cell response [37, 38]. Phosphorylation of STAT3, a marker of the activation of STAT3 pathways, has been reported to be activated mainly by IL-6, which induces Tfh differentiation and IL-21 production [38, 39]. In the present study, we observed significantly up-regulated expression of pSTAT3 in peripheral Tfh cells, as expected after being stimulated with IL-6, and significantly increased serum IL-6, especially in patients with active RA. Meanwhile, there was positive correlation between pSTAT3 expression and serum IL-6, which was consistent with the research of Deng et al. [40]. Further analysis revealed that pSTAT3 expression was positively correlated with circulating Tfh cell frequency, but there was no correlation between pSTAT3 expression and Tfh/Tfr ratio. Taken together, these results demonstrated that elevated serum IL-6 might be pivotal in promoting the imbalance of Tfh and Tfr cells in patients with RA, via activation of the STAT3 signaling pathway by induction of pSTAT3, so as to participate in the development and progression of RA.

Of note, although positive correlation was identified between the expression of pSTAT3 and circulating Tfh cell frequency, there was no correlation between pSTAT3 expression and PD-1, ICOS, or IL-21 expression. These results suggested that the up-regulated expression of pSTAT3 might mainly contribute to the increase in Tfh cell frequency but had no obvious effects on the expression of PD-1, ICOS, or IL-21 in Tfh cells in RA.

Despite all the important findings of this study, there are some limitations as well. In addition to PD-1 and ICOS, the chemokine receptors CXCR3 and CCR6 are also commonly used markers that define Tfh subsets, namely Tfh1, Tfh2, and Tfh17 [41]. These Tfh subsets are reported to exert different helper functions to B cells, which makes their exploration highly significant; these will be pursued in our future studies.

## Conclusions

To the best of our knowledge, this is the first report to demonstrate the imbalance of Tfh subsets and Tfr cells in patients with RA. Increased Tfh cell percentages and decreased Tfr cell frequencies resulted in elevated Tfh/Tfr ratios. Augmented capacity of the circulating Tfh cells to secrete IL-21 and imbalance of Tfh/Tfr cells might contribute to the breakdown of self-tolerance in RA, and thus serve as a potential target for therapy and a meaningful tool for disease evaluation. Enhanced IL-6/pSTAT3 signaling might be potent in promoting the Tfh/Tfr imbalance, mainly via promotion of Tfh cells, and the immunopathogenesis of RA.

## Additional files

Additional file 1: **Figure S1.** Gating strategy describing the Tfh and Tfr cells. In flow cytometry analysis, the gating strategy describing the Tfh and Tfr cells was as follows: (A) stained blood cells were gated for lymphocytes, which were further gated for CD3^+^ T cells. The CD3^+^ T cells were set to exclude the monocytes and further gated for CD4^+^ T cells. Then CD3^+^CD4^+^ T cells were further divided based on the expression of CXCR5, and CD4^+^CXCR5^+^ T cells were further gated for calculating the expression of PD-1, ICOS, IL-21, and pSTAT3. (B) To define Tfh and Tfr cells, CD4^+^ T cells were gated based on expression of CXCR5 and Foxp3. CXCR5^+^Foxp3^−^ cells were defined as Tfh cells, while CXCR5^+^Foxp3^+^ cells were defined as Tfr cells. The cut off for Foxp3 in CD4^+^ cells was determined based on fluorescence minus one (FMO) and isotype control subjects of Foxp3. (TIF 166 kb)

Additional file 2: **Figure S2.** Gating strategy describing the expression of PD-1, ICOS, IL-21 and pSTAT3 in CD4^+^CXCR5^+^ T cells. CD4^+^CXCR5^+^ T cells were gated for determining the expression of PD-1, ICOS, IL-21, and pSTAT3. The cut off for PD-1 (A), ICOS (B), IL-21 (C), or pSTAT3 (D) positivity in CD4^+^CXCR5^+^ T cells was determined based on fluorescence minus one (FMO) and isotype control subjects of PD-1, ICOS, IL-21, or pSTAT3. (TIF 240 kb)

## Abbreviations

ACPA: anti-citrullinated peptide antibodies
ACR: American College of Rheumatology
AS: Ankylosing spondylitis
CD40L: CD40 ligand
CRP: C-reactive protein
CXCR5: C-X-C chemokine receptor type 5
DAS28: Disease Activity Score in 28 joints
EAMG: Experimental autoimmune myasthenia gravis
EULAR: European League Against Rheumatism
GC: Germinal centers
HCs: Healthy controls
ICOS: Inducible co-stimulator
IL-21: Interleukin-21
IQR: Interquartile range
MG: Myasthenia gravis
MS: Multiple sclerosis
PD-1: Programmed death-1
pSTAT3: Phosphorylated signal transducer and activator of transcription 3
RA: Rheumatoid arthritis
RF: Rheumatoid factor
SLE: Systemic lupus erythematosus
Tfh cell: Follicular helper T cell
Tfr cell: Follicular regulatory T cell
Tregs: Regulatory T cells
