# Supplementary figures and images for: Enhanced IL-6/phosphorylated STAT3 signaling is related to the imbalance of circulating T follicular helper/T follicular regulatory cells in patients with rheumatoid arthritis
Source: Arthritis Res Ther. 2018 Aug 29;20:200. doi: 10.1186/s13075-018-1690-0 (PMC6116385; doi:10.1186/s13075-018-1690-0)

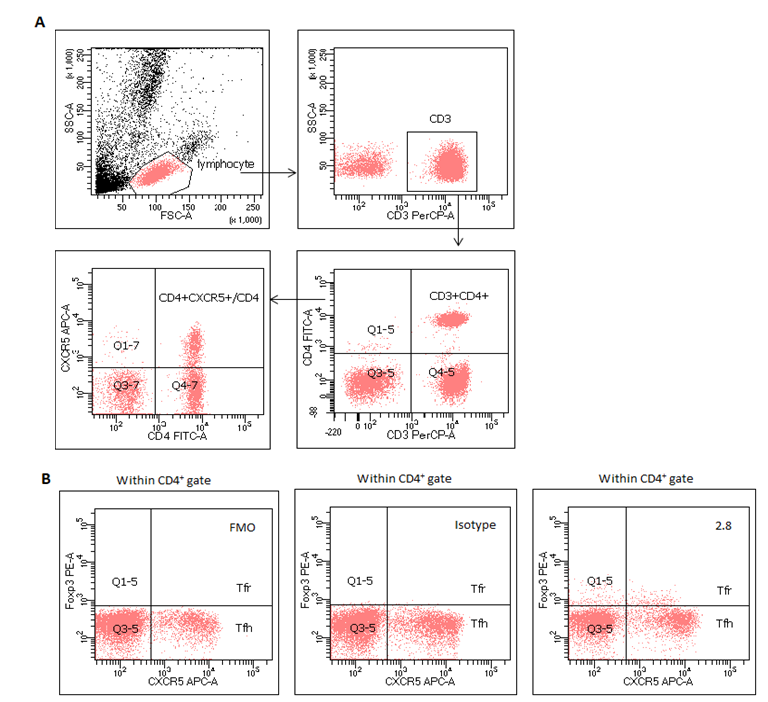

Supplement: Supplementary file 1 — Figure S1. Gating strategy describing the Tfh and Tfr cells. In flow cytometry analysis, the gating strategy describing the Tfh and Tfr cells was as follows: (A) stained blood cells were gated for lymphocytes, which were further gated for CD3+ T cells. The CD3+ T cells were set to exclude the monocytes and further gated for CD4+ T cells. Then CD3+CD4+ T cells were further divided based on the expression of CXCR5, and CD4+CXCR5+ T cells were further gated for calculating the expression of PD-1, ICOS, IL-21, and pSTAT3. (B) To define Tfh and Tfr cells, CD4+ T cells were gated based on expression of CXCR5 and Foxp3. CXCR5+Foxp3− cells were defined as Tfh cells, while CXCR5+Foxp3+ cells were defined as Tfr cells. The cut off for Foxp3 in CD4+ cells was determined based on fluorescence minus one (FMO) and isotype control subjects of Foxp3. (TIF 166 kb) [file 13075_2018_1690_MOESM1_ESM.tif]

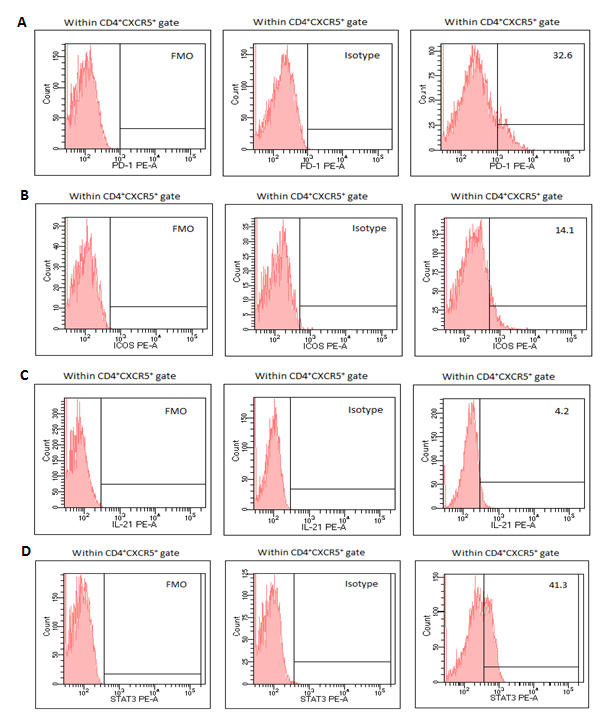

Supplement: Supplementary file 2 — Figure S2. Gating strategy describing the expression of PD-1, ICOS, IL-21 and pSTAT3 in CD4+CXCR5+ T cells. CD4+CXCR5+ T cells were gated for determining the expression of PD-1, ICOS, IL-21, and pSTAT3. The cut off for PD-1 (A), ICOS (B), IL-21 (C), or pSTAT3 (D) positivity in CD4+CXCR5+ T cells was determined based on fluorescence minus one (FMO) and isotype control subjects of PD-1, ICOS, IL-21, or pSTAT3. (TIF 240 kb) [file 13075_2018_1690_MOESM2_ESM.tif]
